# The Gateway Reflex, a Novel Neuro-Immune Interaction for the Regulation of Regional Vessels

**DOI:** 10.3389/fimmu.2017.01321

**Published:** 2017-10-18

**Authors:** Yuki Tanaka, Yasunobu Arima, Daisuke Kamimura, Masaaki Murakami

**Affiliations:** ^1^Molecular Psychoimmunology, Graduate School of Medicine, Institute for Genetic Medicine, Hokkaido University, Sapporo, Japan

**Keywords:** gateway reflex, inflammation amplifier, central nervous system, chemokines, pathogenic CD4^+^ T cells, blood-brain barrier

## Abstract

The gateway reflex is a new phenomenon that explains how immune cells bypass the blood–brain barrier to infiltrate the central nervous system (CNS) and trigger neuroinflammation. To date, four examples of gateway reflexes have been discovered, each described by the stimulus that evokes the reflex. Gravity, electricity, pain, and stress have all been found to create gateways at specific regions of the CNS. The gateway reflex, the most recently discovered of the four, has also been shown to upset the homeostasis of organs in the periphery through its action on the CNS. These reflexes provide novel therapeutic targets for the control of local neuroinflammation and organ function. Each gateway reflex is activated by different neural activations and induces inflmammation at different regions in the CNS. Therefore, it is theoretically possible to manipulate each independently, providing a novel therapeutic strategy to control local neuroinflammation and peripheral organ homeostasis.

## Introduction

The nervous system can sense various environmental stimulations such as light, sound, and temperature through the activation of specific neurons. In addition, events in social interactions that cause psychological alterations such as anxiety, depression, or euphoria can be regarded as environmental stimulations. These stimuli can cause chronic stress that is detrimental to health. A well-defined mechanism is the release of corticosteroid hormones *via* the hypothalamus–pituitary–adrenal grand axis, which systemically modulates immune responses (1, 2). In addition to systemic regulation, there exist local regulations of the inflammatory status by specific neural activations. For example, sensory neural stimulation in the soleus muscles by gravity induces chemokine expressions in the dorsal vessels of the fifth lumbar (L5) but not at other levels of the spinal cord *via* sympathetic (adrenergic) nerve activation (3). In the case of an animal model of multiple sclerosis (MS), experimental autoimmune encephalomyelitis (EAE), chemokine upregulation triggers the infiltration of central nervous system (CNS)-autoreactive CD4^+^ T cells (pathogenic CD4^+^ T cells) from the L5 dorsal vessels into the CNS (3). This unexpected neuro-immune interaction led us to hypothesize that other specific neural stimulations may locally affect immune reactions and organ functions in different ways. Local neuro-immune communications can have pro-inflammatory (3, 4) and anti-inflammatory effects (5–10). In this review, we summarize specific neuro-immune interactions that regulate neuroinflammation and organ homeostasis.

## The Inflammatory Reflex

**Figure 1:**
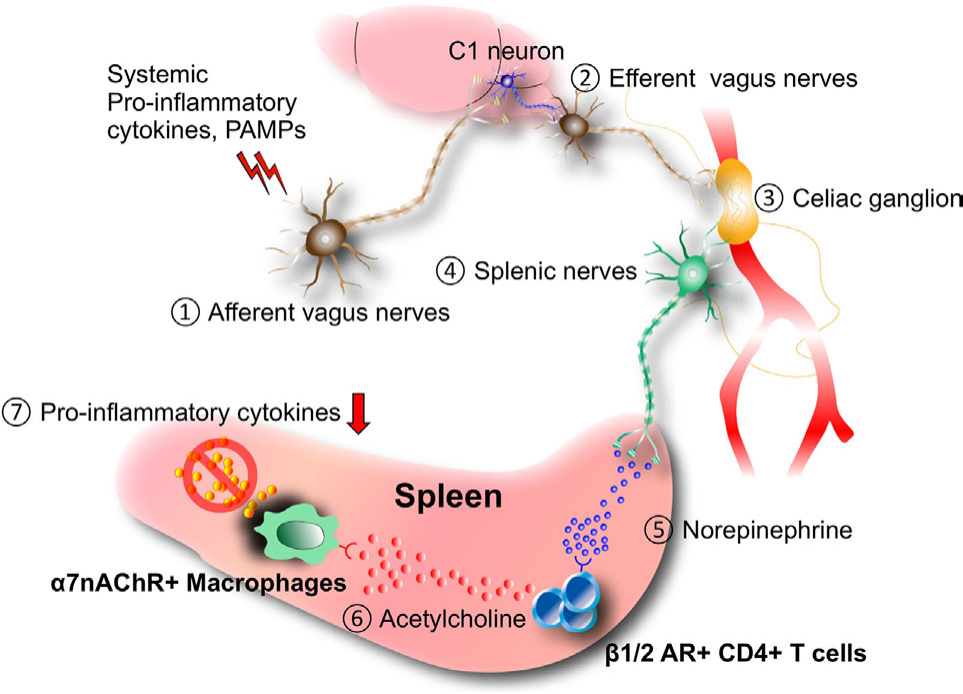
Inflammatory reflex. Afferent (1) and efferent vagus nerve (2) stimulation by systemic pro-inflammatory cytokines and/or pathogen-associated molecular patterns (PAMPs) during septic shock, ischemia–reperfusion injury, and other inflammatory conditions induces neural activation in the celiac ganglion (3), followed by the production of norepinephrine (NE) from the splenic nerves (4). NE (5) stimulates the release of acetylcholine from a novel CD4^+^ T cell subset that expresses choline acetyltransferase and β1/2 adrenergic receptor (AR) (6). Released acetylcholine (6) acts on macrophages expressing α7 nicotinic acetylcholine receptor (α7nAChR) to inhibit the expression of pro-inflammatory cytokines including TNFα. It is suggested that C1 neurons in the medullary reticular formation of the brain mediate the anti-inflammatory effect.

Because clinical studies indicated that nicotine administration or smoking can improve colon inflammation, Dr. Kevin Tracey and his colleagues hypothesized that the parasympathetic (cholinergic) nervous system may regulate an inflammatory response (6, 11–13). Using a murine model of sepsis, they demonstrated that activation of the vagus nerves, which mainly consist of parasympathetic nerves, inhibits systemic inflammation. They termed this neural reflex as the “inflammatory reflex” (14–18). In this example of the inflammatory reflex, lipopolysaccharide (LPS) injection in mice induced norepinephrine (NE) secretion in the spleen through the activation of splenic and vagus nerves. They found a novel subset of CD4^+^ T cells that produce acetylcholine in response to NE. The released acetylcholine was found to act on macrophages that express α7 nicotinic receptor and suppress the LPS-induced inflammatory response (Figure 1) (14). This cascade also promotes anti-inflammatory reactions during ischemia–reperfusion injury (5, 7). A more recent study revealed that direct stimulation of C1 neurons in the medullary reticular formation of the brain conferred the same anti-inflammatory effect (5). It is also reported that stimulating mice by electroacupuncture at the Zusanli acupoint (ST36) located near the common peroneal and tibial branches of the sciatic nerve or by direct stimulation of the sciatic nerve inhibits septic shock through vagal activation and dopamine production (9). Furthermore, local ultrasound also induces the anti-inflammatory splenic neuro-immune interaction (19). Recently, the inflammatory reflex has been tested in a first-in-human trial with promising results (20). These findings solidify a scientific basis for acupuncture and physical therapy.

## The Gateway Reflexes

### Gravity-Gateway Reflex

Since the CNS historically has been considered as an immunologically privileged site due to the blood-brain barrier (BBB) (21), we wondered where and how immune cells invade the CNS to cause neuroinflammation. Through a series of experiments using EAE, we found that regional neural activation by the soleus muscle, which senses gravitational force, determines the location of immune cell entry into the CNS by altering the properties of blood vessels (3). To the best of our knowledge, this was the report that linked gravity and local inflammation through neuro-immune interactions.

The BBB is formed by tight cell-cell interactions between pericytes, endothelial cells, and astrocyte end-feet. Tight junctions are critical for separating the blood and cerebrospinal fluid (22). However, the barrier is not perfect, and it is widely recognized that immune cells can invade the CNS to cause autoimmune diseases such as MS. Recent studies have demonstrated the presence of CNS lymphatic vessels that connect to the cervical lymph nodes and may serve as an exit for immune cells from the CNS (23, 24). It is also known that breaching of the BBB is observed in neurodegenerative diseases such as Alzheimer’s disease and Parkinson’s disease (25). Inflammation is a key component to modulate the integrity of the BBB, and pro-inflammatory cytokines including IL-1β, IL-17A, IFNγ, and TNFα are known to increase BBB permeability (26, 27).

Multiple sclerosis is a chronic inflammatory disease in the CNS that is characterized by impairments in sensory, motor, autonomic, and cognitive functions due to demyelination (28). Genetic factors strongly contribute to the pathogenesis of MS. Genome-wide association studies showed that certain alleles of major histocompatibility complex (MHC) class 2 and genes involved in CD4^+^ T cell activation and survival are genetically associated with MS development (29–33). These genetic data strongly suggest that autoreactive CD4^+^ T cells are essential for the pathogenesis of MS. Animal models of MS including EAE also demonstrated the pivotal role of autoreactive CD4^+^ T cells (3, 4, 34–36). Thus, suppression of the differentiation and activation of CD4^+^ T cells to autoreactive pathogenic T cells or blockade of their entry into the CNS could be a promising therapeutic strategy for MS.

Clinical symptoms of MS are dependent on the location of the demyelination, and MS patients show various damaged CNS sites. Given the wide distribution of a target autoantigen myelin in the white matter of the CNS (28, 33, 37), the heterogeneity of the damaged sites in each patient suggests an unknown mechanism. To identify the initial invasion site(s) of pathogenic CD4^+^ T cells into the CNS, we used an adoptive transfer of CNS-autoreactive CD4^+^ T cells to cause EAE (pathogenic CD4^+^ T cells) and made whole-mount frozen sections of adult mice. These sections were made just before the onset of EAE clinical symptoms to find the first entry site of the pathogenic CD4^+^ T cells. This analysis revealed that pathogenic CD4^+^ T cells mainly accumulated at the dorsal vessels of the L5 spinal cord but not in the upper spinal levels or brain at the preclinical time point. Compared to the dorsal vessels of the L1 spinal cord, many chemokines, including CCL20, which attracts IL-17-secreting type-17 CD4^+^ T (Th17) cells that have a vital pathogenic role in EAE (38, 39), are upregulated in the L5 dorsal vessels. Indeed, the L5 accumulation of pathogenic CD4^+^ T cells was inhibited by anti-CCL20 antibody treatment or by using CCL20 receptor-deficient CD4^+^ T cells. Even without EAE induction, chemokine levels were higher in the L5 dorsal vessels than in the L1 cord, although the levels were lower than those seen in the pathological condition of EAE. These results suggest that L5 dorsal vessels have some unique property under both normal and disease conditions. It is known that the L5 spinal level has the largest dorsal root ganglion (DRG) among spinal levels in both human and mice, and it is reported that L5 DRG neurons are connected to the soleus muscles, which are the main anti-gravity muscles and are activated even in steady state (40, 41). These facts led us to hypothesize that gravity stimulation to the soleus muscles may activate the L5 dorsal vessels to produce the chemokines that form the initial gateway for pathogenic CD4^+^ T cells. We examined this possibility using a ground test employed by the National Aeronautics and Space Administration (42). When normal mice were suspended by tail in a handstand position to free the hind legs from gravity stimulus, the chemokine expressions of the L5 dorsal vessels decreased and pathogenic CD4^+^ T cells failed to accumulate at L5. The cells instead invaded the cervical cords as if another gateway had been formed in response to the greater gravity stimulation on the arm muscles due to the tail suspension. Consistently, the tail suspension significantly decreased the expression levels of c-Fos, a marker for neural activation, in the L5 DRG. In addition, when the soleus muscles of the suspended mice were stimulated by weak electric pulses, chemokine and c-Fos expression levels and pathogenic CD4^+^ T cell accumulation at the L5 dorsal vessels were restored (3). These data suggest that regional sensory neural activation by gravity mediates local inflammation at L5 dorsal blood vessels, representing a novel neuro-immune interaction—the gravity-gateway reflex (14, 16–18, 43–46).

**Figure 2:**
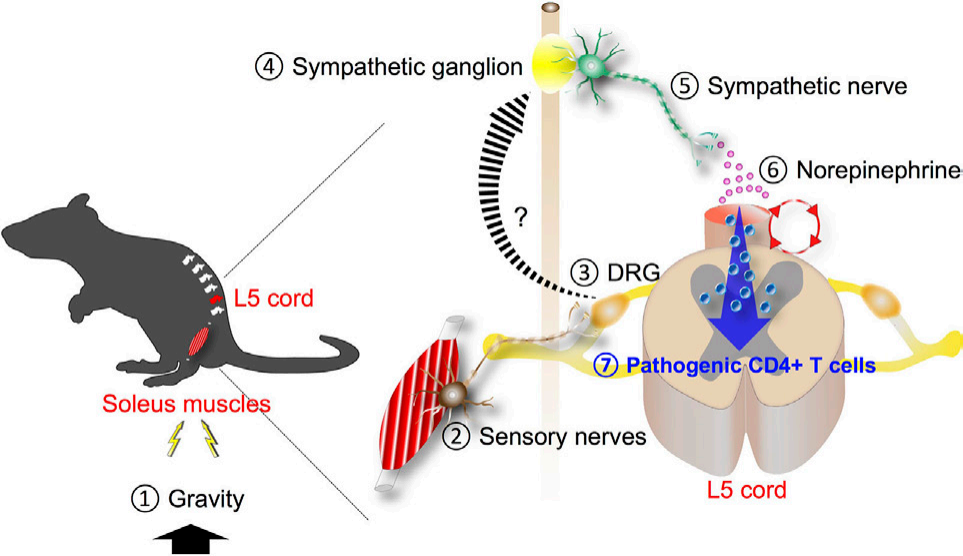
Gravity-gateway reflex. Gravity stimulation (1) induces the activation of sensory nerves in the soleus muscles (2). The cell bodies of these neurons are present at the dorsal root ganglion (DRG) of the fifth lumbar (L5) spinal cord (3). Neural signals through the L5 DRG neurons are transmitted to sympathetic ganglion nearby (4) and activate sympathetic nerves (5), leading to NE secretion (6) at the L5 dorsal vessels. NE boosts the inflammation amplifier (red circle) there, causing chemokine production including CCL20 and recruitment of pathogenic CD4^+^ T cells that express CCL20 receptor, the receptor for CCL20 (7).

Since the autonomic nervous system mainly controls the function of blood vessels, its involvement in the gravity-gateway reflex was suggested. Neural activation based on c-Fos levels was higher in sympathetic ganglions of the L5 level than in those of L1. In addition, after tail suspension, blood flow speed at the L5 dorsal vessels became slower whereas in other blood vessels including L1 dorsal vessels, portal vein, or femoral artery it was not significantly affected. Furthermore, the slowed speed at the L5 dorsal vessels was recovered by electronic stimulation to the soleus muscles. These results suggest that autonomic nerves, particularly sympathetic nerves, could be involved. Functionally, pharmacological blockade of β-adrenergic receptors or chemical sympathectomy inhibited chemokine expressions and pathogenic CD4^+^ T cell accumulation at the L5 dorsal vessels and suppressed the severity of EAE (3). Thus, the gravity-gateway reflex involves local sensory-sympathetic communications for the gateway formation at the L5 dorsal vessels (Figure 2). These results represented the first example of a local neuro–immune interactions that regulate the condition of specific blood vessels to promote chemokine expression. Moreover, because gravity is an inevitable stimulus to land animals, the gravity-gateway reflex may have a physiological role that we have acquired during evolution. It also bears consideration for the health of astronauts and future space exploration.

### Electric-Gateway Reflex

**Figure 3:**
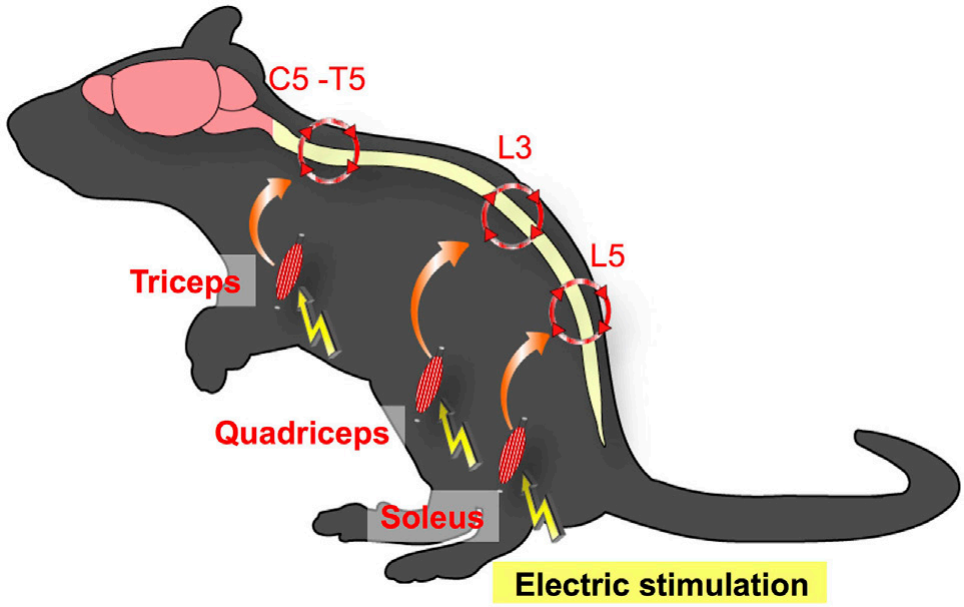
Electric-gateway reflex. Neural activation using weak electric stimulation can induce the gateway reflex. Electric stimulation to the triceps induces chemokine upregulation at the dorsal vessels of the fifth cervical (C5) to fifth thoracic (T5) spinal cord *via* the inflammation amplifier activation (red circles). Likewise, electric stimulation of the quadriceps triggers chemokine upregulation at the L3 dorsal vessels, whereas the L5 gateway is formed by electric stimulation to the soleus muscles.

We next examined whether the gateway reflex is a specific property of the soleus–L5 axis and whether it can be artificially manipulated. We found that weak electric stimulation of neurons in different muscles of mice can create gateways at different levels of the spinal cord. For example, electric stimulation of the quadriceps, which are controlled by L3 DRG neurons, induced chemokine expressions at the L3 dorsal vessels. Moreover, stimulation of the forefoot muscles upregulated chemokines levels at the dorsal vessels in the cervical to thoracic spinal cords (Figure 3) (3). We termed this artificial control of the immune cell gateway in the BBB as the electric-gateway reflex. These results indicate that the gateway reflex can be artificially controlled and suggest a possible therapeutic target for CNS inflammatory diseases such as MS.

### Pain-Gateway Reflex

**Figure 4:**
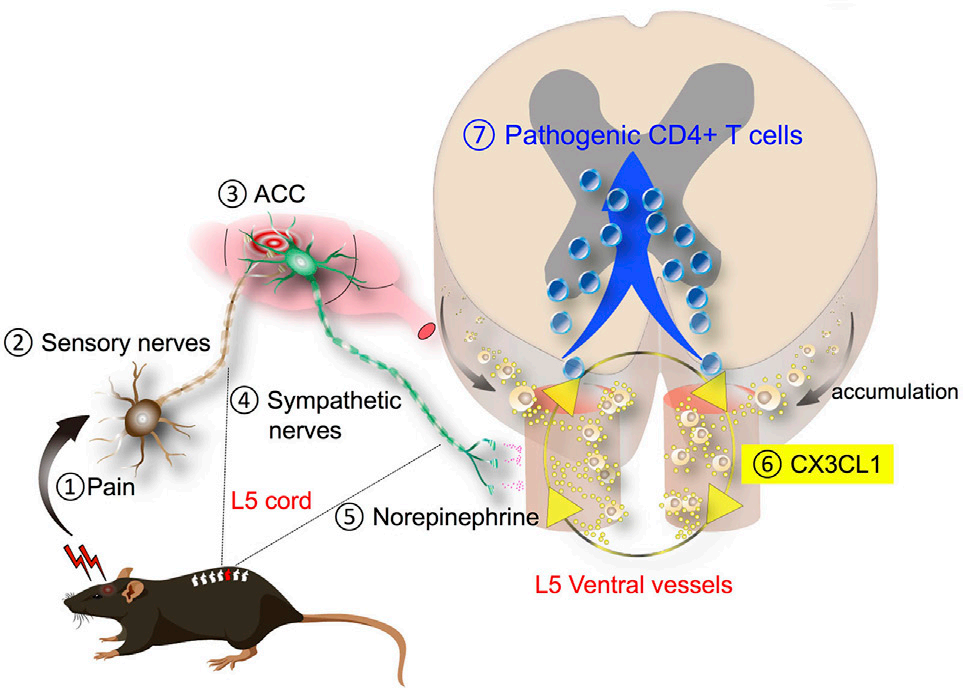
Pain-gateway reflex. Pain-induced sensory stimulation (1, 2) induces the activation of the anterior cingulate cortex (ACC), a pain-processing region in the brain (3), which leads to the activation of specific sympathetic nerves (4), followed by norepinephrine (NE) secretion around the ventral blood vessels of the spinal cords (5). Because major histocompatibility complex (MHC) class 2^Hi^CD11b^+^ monocytes are most abundant in the fifth lumbar spinal cord (L5), the L5 region is mainly affected after pain induction. NE secreted around the ventral blood vessels stimulates the production of chemokine CX3CL1 from MHC class 2^Hi^CD11b^+^ monocytes. Because MHC class 2^Hi^CD11b^+^ monocytes express CX3CL1 receptor CX3CR1, a positive loop is formed to recruit more of them (6). MHC class 2^Hi^CD11b^+^ monocytes have an ability to antigen presentation to pathogenic CD4^+^ T cells, leading to their invasion (7) and subsequently cause disease relapse.

We also investigated other sensory inputs on the gateway reflex. We focused on pain sensation because it is a tonic sensory stimulation (47, 48) and a common undesirable symptom that significantly compromises the quality of life in various diseases (49). It is reported that disease severity and pain occurrence in MS are positively correlated (50–52), and a change in pain sensation is described during EAE (53). In the adoptive transfer EAE model, we used recipient mice develop paralysis around 10 days after the transfer of pathogenic CD4^+^ T cells and then recover from EAE symptoms thereafter. To investigate how pain sensation affects EAE symptoms, we induced pain sensation in mice by surgical ligation of the middle branch of the trigeminal nerves, which are exclusively composed of sensory nerves (54). The pain induction at the time of pathogenic CD4^+^ T cell transfer significantly deteriorated EAE symptoms. By contrast, treatment with pain medicines inhibited EAE development (4). Thus, pain is not only an index of the disease status but also plays a pathogenic role during EAE development. Since many MS patients show relapse, and MS patients showing higher disease scores more frequently claim pain (50, 51), we examined the impact of pain induction during a remission phase of EAE. As expected, mice recovered from EAE clearly relapsed into paralysis upon trigeminal nerve ligation as well as by injection of pain-causing chemicals such as substance P and capsaicin (4). As described earlier, the initial gateway is the dorsal vessels of the L5 spinal cord during the first episode of EAE (3). To identify the immune cell gateway during the pain-induced relapse, we performed immunohistological examination of EAE mice in remission phase (EAE-recovered mice). Although the appearance and motility of EAE-recovered mice were indistinguishable from normal healthy mice, high numbers of periphery-derived activated monocytes expressing high levels of MHC class 2 (MHC class 2^Hi^CD11b^+^ cells) were found around the meningeal region of the L5 cord. Interestingly, pain induction directed these cells to the ventral but not dorsal vessels of the L5 cord. Furthermore, activation of NE signal transduction was evident around the L5 ventral vessels, and MHC class 2^Hi^CD11b^+^ monocytes secreted CX3CL1 following NE stimulation at least *in vitro*, suggesting an autocrine/paracrine loop for MHC class 2^Hi^CD11b^+^ monocyte accumulation. Following the monocyte accumulation at the L5 ventral vessels, pathogenic CD4^+^ T cells in the blood flow invaded from the vessels to the spinal cord parenchyma. Thus, L5 ventral vessels are the gateway during pain-induced relapse in the EAE model (Figure 4). The pain-induced EAE relapse can be inhibited by genetic or pharmacological suppression of the pain sensory pathway or by chemical ablation of the sympathetic nerves, suggesting the involvement of a sensory–sympathetic communication akin to the gravity-gateway reflex (4). We hypothesize that systemic hormonal stress responses do not contribute to the pain-gateway reflex because transient stress loading by immobilization or by forced swimming did not induce EAE relapse despite equivalent levels of serum corticosterone, NE, and epinephrine to those induced by the pain model (4). These results suggest that various sensory stimuli trigger gateway reflexes at different regions of the CNS and implicate that pain control may be beneficial for preventing the relapse of neuroinflammation.

### Stress-Gateway Reflex

It is well known that chronic stresses exacerbate illness. Chronic stress conditions often cause gastrointestinal (GI) diseases *via* the brain–gut axis. However, the molecular mechanism remains poorly understood. Because stresses are associated with neural activation involving brain regions such as the paraventricular nucleus (PVN), the dorsomedial nucleus of hypothalamus (DMH), the dorsal motor nucleus of the vagus nerve (DMX), and the vagal nerve pathway (55), we hypothesized that chronic stresses might activate another specific gateway reflex in the brain. Under chronic stress conditions such as sleep disturbance, we serendipitously found that EAE caused severe GI dysfunction with high mortality (56). While donor pathogenic CD4^+^ T cells accumulated at the L5 dorsal vessels under normal condition due to the gravity-gateway reflex (Figure 2), the stress condition directed them to invade at specific vessels of the boundary area to establish brain microinflammation. Therefore, chronic stresses can change the immune cell gateway from L5 to brain. Indeed, chemokine expressions in the specific vessels of the boundary area of the third ventricle (3V), dentate gyrus, and thalamus increased, whereas those in the L5 dorsal vessels decreased in EAE mice under chronic stress (56).

**Figure 5:**
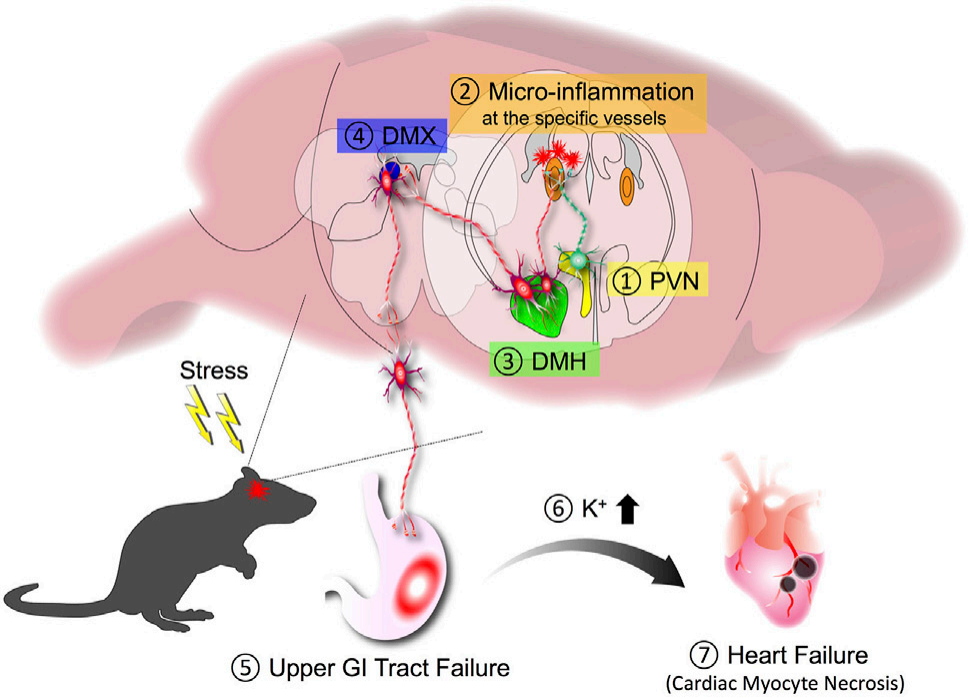
Stress-gateway reflex. Under a chronic stress condition, experimental autoimmune encephalomyelitis mice showed high mortality that was associated with severe gastrointestinal (GI) failure. Chronic stress induces activation of the paraventricular nucleus (PVN) (1), which activates neurons connecting to the specific blood vessels of the boundary region of the third ventricle, dentate gyrus, and thalamus to establish microinflammation in the brain (2). The resulting microinflammation activates a resting neural pathway to the dorsomedial nucleus of hypothalamus (DMH) (3) and dorsal motor nucleus of the vagus nerve (DMX) (4) to cause severe upper GI tract failure (5). The upper GI tract failure results in the increase of potassium ions in the blood (6), which finally causes heart failure with cardiac myocyte necrosis (7).

The resulting microinflammation in the brain specifically enhanced a novel neural pathway that includes the PVN, DMH, and vagal neurons. Neural tracing revealed neural connections, particularly TH^+^ noradrenergic connections, from the PVN to the specific vessels and from the specific vessels to the DMH, which does not consist of TH^+^ connections. Since the PVN is a principal integrator of stress signals, activation of the PVN is expected to influence specific blood vessels *via* the new identified neural circuit. Chemokine expressions including CCL5 at the specific vessels were upregulated in mice with stress only. In the presence of pathogenic CD4^+^ T cells in stressed mice, these cells detect the chemokine upregulation, causing brain microinflammation at the specific vessels of the boundary region of the 3V, dentate gyrus, and thalamus, followed by the accumulation of various immune cells including periphery-derived MHC class 2^Hi^CD11b^+^ cells (56). It is well known that an inflammatory response involves the production of various substances such as ATP that can serve as both inflammatory mediator and neurotransmitter (57, 58). We therefore tested an ATP receptor antagonist injected at the specific blood vessels of the boundary area of the 3V, dentate gyrus, and thalamus and found that neural activation in the DMH region was clearly inhibited, with a significant improvement in the mortality rate of EAE mice with stress. Furthermore, inhibition of the brain microinflammation by cytokine neutralization or blockage of the neural pathway also suppressed the GI dysfunction and improved mortality (56). We examined whether microinflammation at the specific blood vessels is sufficient for stress–EAE phenotypes. Importantly, direct injection of cytokines or ATP at the specific vessels established severe GI failure in mice with stress. These results suggest that microinflammation at the specific vessels turns a resting neural pathway on *via* ATP production, and the ATP-induced neural activation to the DMH region strongly enhances the stress response to cause severe GI damages *via* the DMX (Figure 5). These results reveal a direct association between brain microinflammation and GI homeostasis through a newly established specific neural pathway under chronic stress conditions (56). We defined this phenomenon as the stress-gateway reflex. Several studies suggest a cooccurrence of MS and inflammatory bowel diseases (59–63). Moreover, microinflammations in the brain can be observed in patients with neurodegenerative diseases such as Parkinson’s disease, Alzheimer’s disease, non-Alzheimer type dementia (64, 65), epilepsy (66), and psychological disorders (67). In addition, cerebral microbleeding is known to be a risk factor for dementia (68). We therefore suggest that brain microinflammations could act as a switch to turn on new neural pathways to control organ homeostasis including the brain itself, that several comorbidities might be influenced by the presence of brain microinflammation observed in many diseases, and that circulating CD4^+^ T cells can be a biomarker and therapeutic target for stress-induced organ dysfunction.

**Figure 6:**
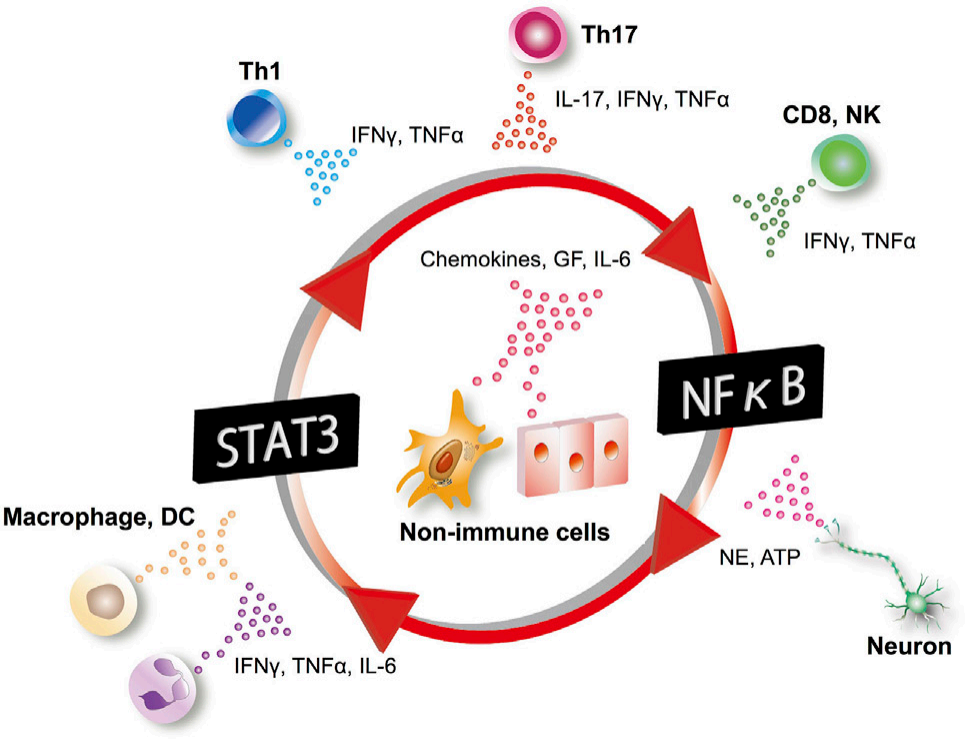
Inflammation amplifier. In the inflammation amplifier, a concurrent activation of the transcription factors NF-κB and STATs in non-immune cells including vascular endothelial cells and fibroblasts induces a multiplier effect on the production of growth factors (GF), chemokines, and cytokines such as IL-6. Various factors activating NF-κB and STATs can operate the amplifier. IL-6 can act on non-immune cells to form an amplifying loop. Excessive production of chemokines and GF through activation of the inflammation amplifier play a key role in the pathogenesis of many chronic inflammatory diseases. DC, dendritic cells; NK, natural killer cells; NE, norepinephrine; Th, helper T cells.

In the gateway reflexes, neural activations induce chemokine production from vascular endothelial cells using the inflammation amplifier, a mechanism in which the concomitant activation of NF-κB and STATs in non-immune cells, such as fibroblasts and endothelial cells, leads to the hyperactivation of NF-κB (Figure 6). The inflammation amplifier is involved in the pathogenesis of several disease models in mice, and evidence of its activation has been demonstrated in human clinical specimens (69–74). Since neurotransmitters such as NE can promote NF-κB activity (75), they enhance the amplifier to further induce NF-κB target genes including chemokines (Figure 6) (3). Further details about the inflammation amplifier can be found in other review articles (69, 76).

## A Myeloid Subset That Mediates the Gateway Reflex

The gateway reflex suggests artificial stimulation of the appropriate neurons can have a potential clinical application. Analogously, vagal nerve stimulation has been tested to modulate the inflammatory reflex in rheumatoid arthritis patients with positive results (20). Another possibility would be to target specific cell type(s) involved in the gateway reflex. During the study of the pain-gateway reflex, we found that periphery-derived MHC class 2^Hi^CD11b^+^ monocytic myeloid lineage cells play an essential role in pain-induced EAE relapse (Figure 4), suggesting that these are a possible cellular target to control the gateway reflex. Local depletion of MHC class 2^Hi^CD11b^+^ monocytes in the CNS significantly suppressed EAE relapse by pain sensation. Systemic depletion of pathogenic CD4^+^ T cells in EAE-recovered mice also inhibited relapse of EAE, but MHC class 2^Hi^CD11b^+^ monocytes still accumulated around the ventral vessels of the L5 cord. These data indicate that the migration of MHC class 2^Hi^CD11b^+^ monocytes to the L5 ventral vessels by the pain-gateway reflex precedes pathogenic CD4^+^ T cell invasion and is a critical step for the relapse of EAE (4). Experiments using parabiosis showed that MHC class 2^Hi^CD11b^+^ monocytes are derived from the periphery, infiltrate the CNS during the first symptom of EAE, and have long life span in the CNS. Interestingly, the pharmacological blockade of *N*-methyl-d-aspartic acid (NMDA) receptor at the anterior cingulate cortex, where the pain-mediated sensory signal transits to a sympathetic signal, inhibited the pain-induced accumulation of MHC class 2^Hi^CD11b^+^ monocytes in EAE-recovered mice, while activating this neural pathway by the injection of a NMDA receptor agonist induced the accumulation of the cells even without pain induction (4). Similar periphery-derived MHC class 2^Hi^CD11b^+^ monocytic cells were also detected in the specific vessels of EAE mice under stress conditions (56). These results suggest that periphery-derived MHC class 2^Hi^CD11b^+^ monocytic cells are a unique myeloid subset that serves as an interface for neuro-immune interactions during the gateway reflex and is a potential cellular target for the treatment of inflammatory diseases in the CNS.

## Perspective

Growing evidence has demonstrated significant functions of specific regional neuro-immune interactions during inflammation and disease. As described in this review, vagus and splenic nerve-mediated control of specific subsets of CD4^+^ T cells and macrophages that produce and respond to acetylcholine is a main axis of the inflammatory reflex (14–18, 77). For the gateway reflexes, different players including CNS-reactive pathogenic CD4^+^ T cells and periphery-derived MHC class 2^Hi^CD11b^+^ monocytic cells drive the response (3, 4, 44, 45, 56). Recently, a pilot study was performed that stimulated the vagus nerve of seven Crohn’s disease patients, and five patients showed deep remission at 6 months of follow-up without major side effects (78). In addition, Koopman et al. showed that vagus nerve stimulation in rheumatoid arthritis patients significantly improved disease severity with reduced TNF production (20). To activate specific neurons from the body surface to induce desired effects, elucidation of the precise neural networks for both reflexes is anticipated. Although identification of the detailed neural pathways is challenging, recently developed imaging techniques that use a tissue decoloring reagent, CUBIC (79–81), in conjunction with various neural tracers and transgenic mice will help. Further investigation of the gateway reflexes in response to various stimuli will also help identify novel functional neural connections that govern organ homeostasis or pathogenesis associated with local inflammation. Mapping of the neural connections and identifying more examples of gateway reflexes could lead to the elucidation of physiological functions of the gateway reflexes, which are currently not clear. In addition, whether the MHC class 2^Hi^CD11b^+^ monocytic lineage and brain microinflammation-inducing CD4^+^ T cells are present in humans is an unanswered question. To do so, specific markers for these cell types are needed.

Because neuronal circuits run throughout the whole body and because immune cells (10, 82–84) and non-immune cells (3, 4, 56) can secrete and respond to neurotransmitters, specific local neuro-immune interactions such as the gateway reflex and inflammatory reflex have tremendous therapeutic potential for the treatment of various diseases without any major systemic side effects.

## Author Contributions

All authors wrote and elaborated manuscript and figures.

## Conflict of Interest Statement

The authors declare that the research was conducted in the absence of any commercial or financial relationships that could be construed as a potential conflict of interest.
